# Relationship between back posture and early orthodontic treatment in children

**DOI:** 10.1186/s13005-021-00255-5

**Published:** 2021-02-05

**Authors:** Isa Klostermann, Christian Kirschneck, Carsten Lippold, Sachin Chhatwani

**Affiliations:** 1 Department of Orthodontics, University of Muenster, Waldeyerstraße 30, 48149 Muenster, Germany; 2 Department of Orthodontics, University Medical Centre of Regensburg, Franz-Josef-Strauss-Allee 11, 93053 Regensburg, Germany; 3 Department of Orthodontics, School of Dentistry, Faculty of Health, Witten/Herdecke University, Alfred-Herrhausen Str. 45, 58455 Witten, Germany

**Keywords:** Early orthodontic treatment, Body posture, Rasterstereography, Fränkel type II appliance

## Abstract

**Background:**

The purpose of this study was to analyze the relationship between body posture and sagittal dental overjet in children before and after early orthodontic treatment with removable functional orthodontic appliances.

**Methods:**

Angle Class II patients (mean age 8.2 ± 1.2 years; 29 males and 25 females) with a distinctly enlarged overjet (> 9 mm) were retrospectively examined regarding body posture parameters before and after early orthodontic treatment. In addition, changes in overjet were investigated with the aid of plaster models. Forms of transverse dysgnathism (crossbite, lateral malocclusions) and open bite cases were excluded. Body posture parameters kyphosis, lordosis, surface rotation, pelvic tilt, pelvic torsion and trunk imbalance were analyzed by means of rasterstereographical photogrammetry to determine, if the orthodontic overjet correction is associated with specific changes in posture patterns.

**Results:**

In nearly all patients an overjet correction and an improvement regarding all body posture and back parameters could be noted after early orthodontic treatment. Overjet reduction (− 3.9 mm ± 2.1 mm) and pelvic torsion (− 1.28° ± 0,44°) were significantly (*p* < 0.05) and moderately correlated (R = 0.338) with no significant associations found for the other posture and back parameters (*p* > 0.05).

**Conclusion:**

Overjet reduction during early orthodontic treatment may be associated with a detectable effect on pelvic torsion.

## Background

A correlation of body posture and craniofacial morphology has been the focus of investigation in many studies. Especially since the 1980’s publications about this topic have increased [1]. Some studies have shown some, albeit sometimes minor, influences [2–6], while other studies have found no impact of orthodontics on body posture [7–11].

The subject of these studies becomes increasingly more important considering interdisciplinary treatment combining orthopedics and orthodontics [4]. Because of the functional connection between the stomatognathic system and the cervical spine, these two fields of medicine are inevitably linked together [12].

Early treatment in children with severe malocclusion, especially of Angle Class II type, could not only prevent incisor trauma, but also have a positive influence on potential orthopedic malformations [13]. There is no general need for patients with Angle Class I, II, III malocclusion to be treated interdisciplinarily. Only patients with an asymmetry of the jaw should undergo interdisciplinary treatment [5].

A growing number of patients with spinal deformities seek orthodontic treatment to enhance body posture [11]. But due to currently lacking evidence regarding associations between disorders of the masticatory system and postural imbalances, patients should avoid irreversible and expensive treatments, if these treatments are aimed at correcting postural imbalances or spinal curvature alteration [14].

Different devices and procedures are used to investigate correlations between the masticatory system and body posture like postural platforms, rasterstereography, surface electromyography and kinesiography [7]. März et al. found some indications of a relationship when investigating 44 patients in seven different mandible positions and optically scanning body posture with the Diers formetric 4D system [15]. The observed different posture parameters could have arisen due to a neuromuscular compensation mechanism [7, 15]. Due to current shortcomings in scientific evidence, this topic is very interesting, considering the fact that changes in posture or the craniomandibular system may affect each other [16].

Deformities of interest in interdisciplinary orthodontic and orthopedic treatment are especially the two most frequent spinal diseases in form of scoliosis and Scheuermann’s disease that in particular arise during childhood [17, 18]. Radiographs were used in the past for the measurement of scoliotic deformities, but due to the required amount of X-ray images during this procedure and thereby high radiation exposure of the patient, video rasterstereography became a popular alternative. This technique was developed in the 1980’s by Hierholzer and Drerup and is a three-dimensional analysis of the back surface. With only one measurement, a 3D footage of the patients’ back can be analyzed and also edited digitally. Therefore it is a useful addition for long-term controls for patients with spine deformities [19].

The aim of this study was to analyze the relationship between sagittal back contour, posture and craniofacial parameters in children before and after early orthodontic treatment with removable appliances with optical 3D back shape measurements.

As a null hypothesis it assumed that there is no difference in body posture before and after early orthodontic treatment with removable appliances.

## Methods

Sample size was determined a priori with G*Power (Heinrich Heine University, Duesseldorf, Germany) for an effect size of 0.5, an alpha level of 0.05 and a power of 80%. The calculation showed that a minimum of 28 patients was needed for this study.

The procedure of optical spine analysis is based on footage of the back with simultaneous projection of stripes onto the back surface. Due to the curvature of the projected stripes, a pattern arises and with three anatomically fixed points, the vertebra prominens and a dimple at the left and right side at the height of the sacrum, a model of the back can be generated using a triangulation method [20–22]. The digitally plotted model is like a visual plaster cast of the back of the patient.

Fifty- four children (29 male, 25 female) at the age of 4.3–10.7 years, who were treated between 2008 and 2017 in an orthodontic practice and presenting mandibular retrognathia (Angle-Class II, corresponding leading symptoms), were retrospectively analyzed before (T1) and after (T2) early orthodontic treatment using rasterstereography (Diers formetric 4D, Diers International, Schlangenbad, Germany). Patients with syndromes, cleft lip and or cleft palate, forms of transverse dysgnathism (crossbite, lateral malocclusions), open bite and long-term medication or systemic diseases like diabetes mellitus were excluded. Patients with significant obesity were also excluded because of limitations regarding the rasterstereographic measurement method applied.

**Fig. 1:**
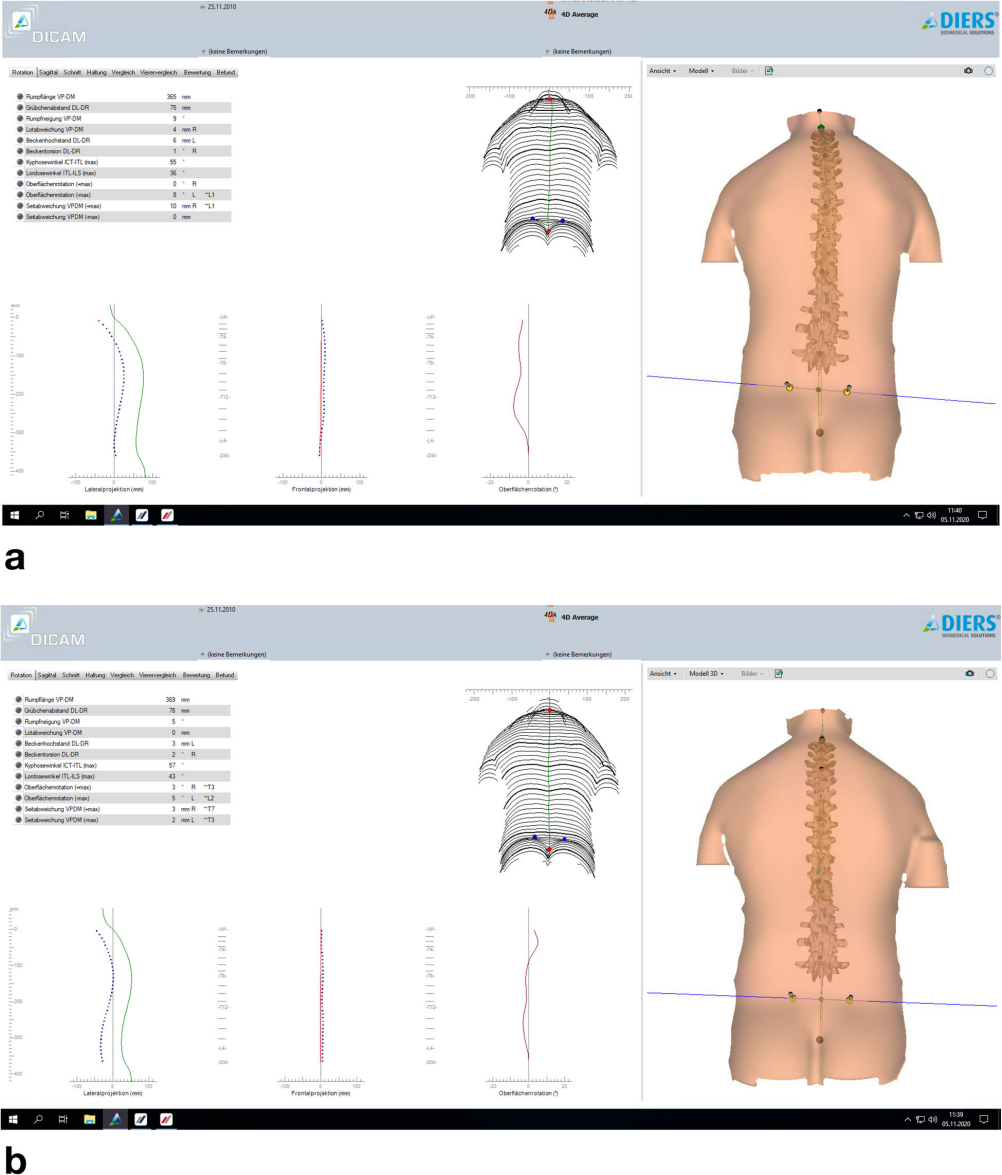
Rasterstereographic measurement of a sample patient before (**a**) and after (**b**) early orthodontic treatment

During rasterstereographic measurement (Fig. 1), the patients stood in a relaxed position and did not pause breathing, so that the individual position could be measured. They were barefoot and only wearing undergarment, thus there were no disturbing influences by clothing. Further details regarding the procedure of rasterstereography used in this study and determined outcome parameters can be found in a pilot study by März et al. [15]. The parameters kyphosis, lordosis, surface rotation, pelvic tilt, pelvic torsion, trunk imbalance were examined to analyze posture and back shape before (T1) and after (T2) early orthodontic treatment.

Incisor overjet was investigated with the aid of plaster models and a manual caliper (Muenchner model, Dentaurum, Ispringen, Germany) at treatment times T1 and T2, in the sagittal plane of the most prominent labial upper incisor as described in Meštrović et al. [23].

**Fig. 2:**
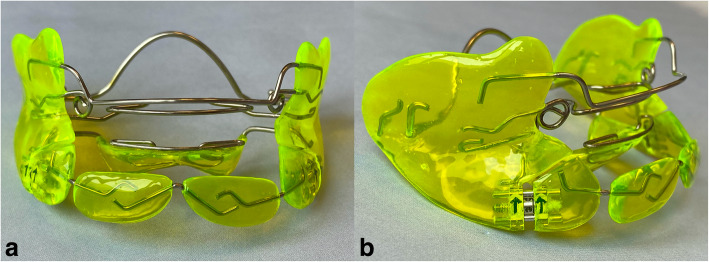
**a**, **b**: Fränkel type II appliance **a**) frontal view **b**) lateral view (Photos by Prof. Dr. Carsten Lippold)

Early orthodontic treatment was performed with a Fränkel type II removable appliance (Fig. 2 a, b). Patients were instructed to wear the appliance at least for 3 h a day and full time at night. The effectiveness of the Fränkel type II appliance in early orthodontic treatment has been shown before [24].

Statistical analysis was performed using the program IBM® SPSS® Statistics 26 (IBM, Armonk, NY, USA). Descriptive-exploratory data analysis was conducted to calculate the arithmetic mean (M), standard deviation (SD), 95% confidence interval (CI), minimum (Min) and maximum (Max). For posture and back parameters the median (MD) was calculated with 95% CI. Normal distribution of the individual parameters was assessed by using Kolmogorov-Smirnov test. For analytical-statistical data analysis of paired samples, non-parametric, dependent Wilcoxon signed ranks tests were used. To check for possible correlations between the degree of overjet change (reduction) and posture and back parameters, a two-sided correlation analysis according to Spearman was conducted, where R > 0.5 / 0.3 / 0.1 according to Cohen [25] corresponds to a strong, moderate or low correlation. The significance level (α-error) was set to *p* ≤ 0.05.

The study was approved by the local ethics committee in Münster, Westfalen-Lippe, Germany with the registration number 2018–340-f-S.

## Results

Mean patient age (± SD) at the time of measurement T1 was 8.2 ± 1.2 years and at the end of treatment T2 10.1 ± 1.3 years. Mean treatment time of early orthodontic treatment was 1.8 ± 0.5 years (Table 1).

**Table 1:** Descriptive statistics of age and overjet at T1, T2 and changes from T1 – T2

Age in years	n	M	SD	95% CI (low – high)	MD	25% Perc	75% Perc	Min	Max
T1	54	8.2	1.2	7.9–8.6	8.3	7.3	9.2	4.3	10.7
T2	54	10.1	1.3	9.7–10.4	10.1	9.0	10.9	6.1	13.1
treatment time T1 – T2	54	1.8	0.5	1.7–1.9	1.8	1.6	1.9	0.8	3.5
Overjet in mm	n	M	SD	95% CI (low – high)	MD	25% Perc	75% Perc	Min	Max
T1	54	11.1	1.8	10.6–11.6	10.5	10.0	12.0	8.0	18.0
T2	54	7.2	2.1	6.6–7.7	7.0	6.0	8.5	3.0	14.0
overjet correction T1 – T2	54	−3.9	2.1	−4.5 – −3.3	−3.5	−4.6	−2.5	−11.0	0.5

*M* arithmetic mean*, SD* standard deviation*, CI* confidence interval*, MD* Median*,* Perc *Percentile, Min* Minimum*, Max* Maximum

Mean overjet measured at the beginning of treatment (T1) was 11.1 mm ± 1.8 mm. From T1 to T2 mean correction of overjet was − 3.9 mm ± 2.1 mm. This led to a mean overjet of 7.2 mm ± 2.1 mm at the end of early orthodontic treatment T2 (Table 1). In one patient case overjet increased by 0.5 mm from 9.5 mm to 10 mm.

Descriptive statistics of back and posture parameters at T1, T2 and of their respective changes from T1 to T2 can be seen in Table 2.

**Table 2:** Descriptive statistics of posture and back parameters at T1, T2 and their respective changes from T1 to T2

*Posture/back parameter*	*n*	*M*	*SD*	*MD*	*95% CI (low – high)*	*Min*	*Max*
kyphotic angle in ° at T1	54	42.89	0.94	42.5	40.0–45.0	28.0	61.0
kyphotic angle in ° at T2	54	42.2	0.79	42.0	41.0–45.0	27.0	54.0
kyphotic angle change in ° from T1 to T2	54	−0.69	0.79	−1.0	− 1.0 – 3.0	−13.0	14.0
lordotic angle in ° at T1	54	35.69	1.03	35.0	32.0–39.0	23.0	48.0
lordotic angle in ° at T2	54	34.17	1.06	33.0	31.0–37.0	19.0	54.0
lordotic angle change in ° from T1 to T2	54	−1.52	0.98	−1.0	−4.0 – 1.0	−19.0	12.0
pelvic tilt in mm at T1	54	3.17	0.39	3.0	3.0–6.0	0.0	15.0
pelvic tilt in mm at T2	54	3.65	0.44	3.0	3.0–6.0	0.0	15.0
pelvic tilt change in mm from T1 to T2	54	−0.74	0.76	0.0	0.0–3.0	−27.0	12.0
pelvic torsion at T1 in °	54	2.46	0.25	2.0	2.0–3.0	0.0	7.0
pelvic torsion at T2 in °	54	2.44	0.23	2.0	2.0–3.0	0.0	6.0
pelvic torsion change in ° from T1 to T2	54	−1.28	0.44	−1.0	−1.0 – 0.0	−11.0	5.0
trunk imbalance in mm at T1	54	6.8	0.86	5.5	3.0–8.0	0.0	32.0
trunk imbalance in mm at T2	54	5.67	0.54	5.0	4.0–7.0	0.0	22.0
trunk imbalance change in mm from T1 to T2	54	−4.91	1.25	−4.5	−8.0 – −1.0	−39.0	17.0
max surface rotation at T1 in °	54	5.52	0.6	5.0	3.0–7.0	0.0	16.0
max surface rotation at T2 in °	54	5.59	0.6	5.0	3.0–7.0	0.0	14
max surface rotation change in ° from T1 to T2	54	−0.41	0.9	0.0	−2.0 – 2.0	−18.0	12.0
min surface rotation in ° at T1	54	5.46	0.67	4.0	3.0–6.0	0.0	25.0
min surface rotation in ° at T2	54	5.31	0.63	4.5	3.0–6.0	0.0	22.0
min surface rotation change in ° from T1 to T2	54	−1.07	0.66	−1.0	−2.0 – 2.0	−15.0	10.0

*M* arithmetic mean*, SD* standard deviation*, MD* Median*, CI* confidence interval*, Min* Miniumum*, Max* Maximum

Kolmogorov-Smirnov tests revealed that not all parameters were normally distributed (*p* < 0.05). Paired, dependent Wilcoxon tests showed that besides age (*p* < 0.001) the amount of overjet measured differed significantly at end of treatment (T2) from the start of treatment (T1) (*p* < 0.001) (Table 3).

**Table 3:** Wilcoxon signed ranks test for back/posture parameters, overjet and age comparing at measurement times T1 and T2

	*kyphotic angle*	*lordotic* *angle*	*pelvic tilt*	*pelvic torsion*	*trunk imbalance*	*max surface rotation*	*min surface rotation*	*overjet*	*age*
Z	−0.954^b^	−1.416^b^	−0.967^c^	− 0.079^b^	−0.887^b^	− 0.031^c^	−0.175^b^	−6.388^b^	−6.396^c^
Asymp. Sig. (2-tailed)	0.34	0.157	0.334	0.937	0.375	0.975	0.861	0.000***	0.000***

Wilcoxon signed ranks test b) based on positive ranks c) based on negative ranks ***) significane at level *p* < 0.001

All postural parameters also changed from T1 to T2: kyphotic angle (− 0.69° ± 0.79°), lordotic angle (− 1.52° ± 0.98°), pelvic tilt (− 0.74 mm ± 0.76 mm), pelvic torsion (− 1.28° ± 0,44°), trunk imbalance (− 4.91 mm ± 1.25 mm), maximum (− 0.41° ± 0.9°) and minimum (− 1.07° ± 0.66°) surface rotation showed a reduction in general, but no significant differences between T1 and T2 (Tables 2 and 3).

**Fig. 3:**
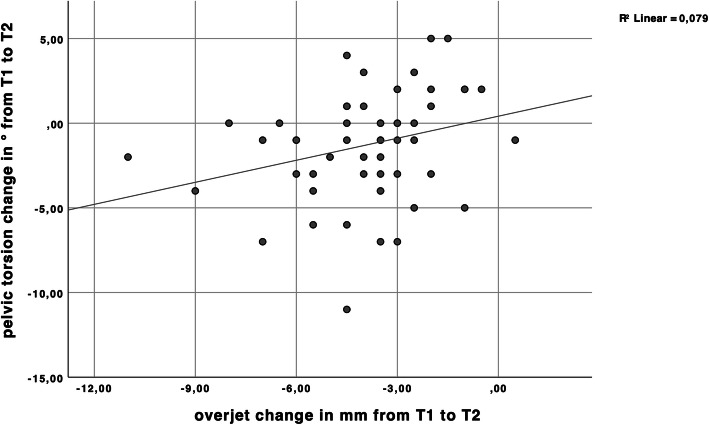
Scatter plot of the correlation between overjet change from T1 to T2 and pelvic torsion change in ° from T1 to T2. Correlation is represented by a linear regression line. R^2^ = 0.079

A significant correlation according to Spearman’s rho correlation test was found between change of overjet from T1 to T2 and pelvic torsion change from T1 to T2 (Table 4, Fig. 3).

**Table 4:** Spearman correlation of change of overjet T1 – T2 and changes of the individual back/posture parameters T1 – T2

*Change of overjet T1 – T2 in correlation with*	*n*	*Correlation Coefficient rho*	*Sig. (2-tailed)*
∆ kyphotic angle T1 – T2	54	0.066	0.634
∆ lordotic angle T1 – T2	54	0.000	0.997
∆ pelvic tilt T1 – T2	54	0.136	0.325
∆ pelvic torsion T1 – T2	54	0.338	0.012*
∆ trunk imbalance T1 – T2	54	−0.148	0.285
∆ surface rotation max T1 – T2	54	0.198	0.151
∆ surface rotation min T1 – T2	54	−0.132	0.343

∆) change of *) Correlation significant at level *p* < 0.05 (2-tailed)

## Discussion

At the beginning of the twenty-first century a huge increase of interest in the topic of dental and orthopedic associations could be noted [1]. Medical awareness increased in recent years. Even though influence of dental occlusion on body balance is debated controversially some authors conclude that the afferent signals of the dental occlusion may have an impact on body balance [26]. Ohlendorf et al. also reported that a temporarily manipulated dental occlusion affects the position of the spine but questioned its clinical effect [27]. Nowadays not only children receive orthodontic treatment, but also adults with spinal abnormities seek help from orthodontists [11]. The aim of this study was to determine, whether different orthopedic posture patterns are associated with a correction of the patients’ overjet by early orthodontic treatment. The method used in this study was rasterstereography with 4D measurement.

During 3D measurements, a recording of the back shape is made in only 0.25 s, comparable to a conventional X-ray image [10]. But even when standing still, involuntary movements of the body like breathing have an impact on the surface of the body and thereby the rasterstereographic measurement. During 4D measurements optional single shootings are made and the impact of involuntary movements can be minimized [21]. An advantage of the 4D measurement is the reliability under dynamic conditions in comparison to a single measurement [19]. It seems that this method is appropriate for assessment of posture and back parameters in this study [28].

Compared to a multicenter, randomized-controlled trial investigating the overall dental and skeletal changes in early orthodontic treatment, the patients in this study were approximately 1.5 years younger (9.7 ± 0.98 years compared to 8.2 ± 1.2 years) [29]. In the same study mean change of overjet was higher − 6.63 mm (95% CI − 7.28 mm – 5.98 mm) than in our study − 3.9 mm (95% CI -4.5 – − 3.3 mm). The appliance used by that study group was a Twin Block appliance which was used in 89 patients [29]. Tulloch et al. showed less overjet reduction than observed in our study after early orthodontic treatment with functional appliances (− 2.66 mm ± 1.81 mm) [30]. A study by Toth and McNamara investigating 40 patients treated with Fränkel type II appliances (− 3.1 ± 1.5 mm) and 40 patients with Twin Block appliances (− 3.6 ± 2.7 mm) showed no significant difference in overjet reduction after 16 months between both appliances [31]. Thus the overjet reduction in this study is in concordance with literature, as well as treatment duration for early orthodontic treatment (1.4 ± 0.57 years) [32]. Probable explanations for discrepancies in results therefore might be differing patient motivation or compliance compared to our study.

This study shows a moderate correlation between sagittal incisor overjet and orthopedic parameters with a significant correlation observed for a reduced overjet and pelvic torsion (*p* < 0,012). Still, the results of this study are in line with other studies that have found no strong connection of back and posture parameters and orthodontic treatment, as no significant differences were found for pelvic torsion from T1 to T2.

Lippold et al. investigated the relationship between orthodpedic findings and craniofacial morphology showing a correlation of craniofacial parameters and thoracic, lordotic and pelvic inclination. They concluded that there is evidence of relations between the body posture in the upper area (cervical to thoracical region) and the craniofacial morphology [4].

In contrast a detectable correlation between dental occlusion and body posture not found by Perinetti [8, 33]. The body’s neuromuscular and anatomical balancing mechanisms may be the reason, why no differences were discovered [8, 15]. It could be that the immediate change in pelvic torsion after overjet correction is an accidental finding, but also it could be thought of that pelvic torsion change plays a role in anatomical balancing.

A study supporting dental and orthopedic associations shows that orthopedic abnormalities are more common in children (3,5–6,8 years) with an Angle class II than in Angle class I. Scoliotic abnormalities are found in 21.1% of children with Angle class II and hypotonic body posture is also common (52.6%). This shows that orthodontic treatment should not be limited to an extremely positioned frontal incisor, but early treatment may also prevent orthopedic misdevelopment in Angle class II dysgnathia [13].

Parrini et al. described modifications of kyphotic angle, upper thoracic inclination and pelvic inclination after 6 months of orthodontic treatment with aligners and thereby showed the influence of orthodontics or change of vertical dimension on body posture [34].

Kamal and Fida have reported that craniocervical posture after Twin Block therapy is more upright [35]. They, however, derived their findings from lateral cephalograms, which did not correspond to natural head position at rest.

The differing results in literature show the importance of further studies to investigate this topic as clear evidence is currently still missing. Future studies should not only investigate immediate effects of orthodontic intervention on body posture, but also possible long-term effects.

Postural control is very complex and is also influenced by visual, vestibular and proprioceptive systems [36]. Due to its multifactorial occurrence it is difficult to emphasize on individual aspects. Further limitations of this study comprise its retrospective and correlative character, which does not allow assessments of causality, as well as the absence of a control group. With regard to overjet correction it can be said that it was not solely of skeletal, but also of dental origin [31]. It would be of interest to investigate the influence of surgical treatment in adults on body posture as the skeletal changes are higher. Future studies should investigate correlations of skeletal changes to body posture especially in the long-term.

Although a minor correlation of overjet reduction and pelvic torsion could be seen in this study the null hypothesis is to be accepted.

## Conclusion

In this study, no significant differences of back and posture patterns were found after early orthodontic treatment with removable appliances. A decrease of overjet was moderately correlated to a change of pelvic torsion. This immediate, but minor presumable influence of orthodontics on orthopedics could lead to further longitudinal studies pursuing this interdisciplinary approach.

## Data Availability

All data is available upon request.
